# Transcriptomic landscapes underlying response and resistance to HDAC inhibitor chidamide in triple-negative breast cancer

**DOI:** 10.1016/j.gendis.2025.101676

**Published:** 2025-05-08

**Authors:** Yanxia Zhang, Yunduo Liu, Mei Zhang, Guanjie Li, Qin Xiang, Shunhong Wu, Keping Xie, Bin Xiao, Linhai Li

**Affiliations:** a Department of Laboratory Medicine, The Affiliated Qingyuan Hospital (Qingyuan People's Hospital), Guangzhou Medical University, Qingyuan, Guangdong 511518, China; b School of Medicine, The South China University of Technology, Guangzhou, Guangdong 510006, China; c School of Public Health, Dali University, Dali, Yunnan 671003, China; d Thyroid and Breast Specialty of General Surgery Area Five, The Affiliated Qingyuan Hospital (Qingyuan People's Hospital), Guangzhou Medical University, Qingyuan, Guangdong 511518, China; e Department of Laboratory Medicine, Southern Medical University Hospital of Integrated Traditional Chinese and Western Medicine, Southern Medical University, Guangzhou, Guangdong 510315, China

Triple-negative breast cancer (TNBC) remains a medical challenge due to limited therapeutics.^1^ Histone deacetylases (HDACs) play vital roles in chromatin remodeling and epigenetics, and their dysregulation is implicated in malignancies, including TNBC.^2^ HDAC inhibitors (HDACis) have shown potent anti-TNBC activity in preclinical studies.^3^ Unfortunately, their clinical applications are beset by drug resistance, about which little is known.^4^ This study systematically explored the clinical significance of HDACs and transcriptomic landscapes underlying response and resistance to HDAC1/2/3/10 selective inhibitor chidamide,^5^ the first approved HDACi for solid tumor treatment, in TNBC.

The expression of 11 HDACs, including chidamide targets HDAC1/2/3/10, was profiled with transcriptome and clinical data of 190 TNBC patients obtained from The Cancer Genome Atlas (TCGA) database (Fig. S1A). HDAC1/2/8/10 showed significant up-regulation in TNBC tissues, whereas HDAC4/5/6/7 were down-regulated (Fig. 1A). Co-expression analysis showed positive correlations between chidamide targets HDAC1/2/3 and other HDACs, including HDAC4/5/6/7/8 (Fig. S1B). Kaplan–Meier analysis indicated that TNBC patients with high HDAC2/4/8/9 expression had better outcomes, while those with high HDAC1/3/5/6/7/10/11 expression had worse outcomes (Fig. S1C). Moreover, positive correlation was found between HDAC1–9 and at least one proliferation marker, including marker of proliferation Ki-67 (MKI67), proliferating cell nuclear antigen (PCNA), and minichromosome maintenance complex component 2 (MCM2) (Fig. S2A). Aberrant oncogene expression and overactive cancerogenic signaling pathways contribute to tumor initiation and progression, prompting further investigation into relationships between HDAC expression and these factors. All 11 HDACs were positively correlated with numerous oncogenes (Fig. S2B), and most HDACs (HDAC1–8) were linked to increased activity in cancer-related signaling pathways, including cell cycle, DNA repair, DNA replication, and estrogen, mitogen-activated protein kinase (MAPK), Notch, or Wnt signal (Fig. S2C). Taken together, considering their abnormal up-regulation in tumors and association with poor prognosis, augmented proliferation, oncogene expression, and cancerogenic signal, HDACs, including most of the chidamide targets, may be promising targets for TNBC.

**Figure 1:**
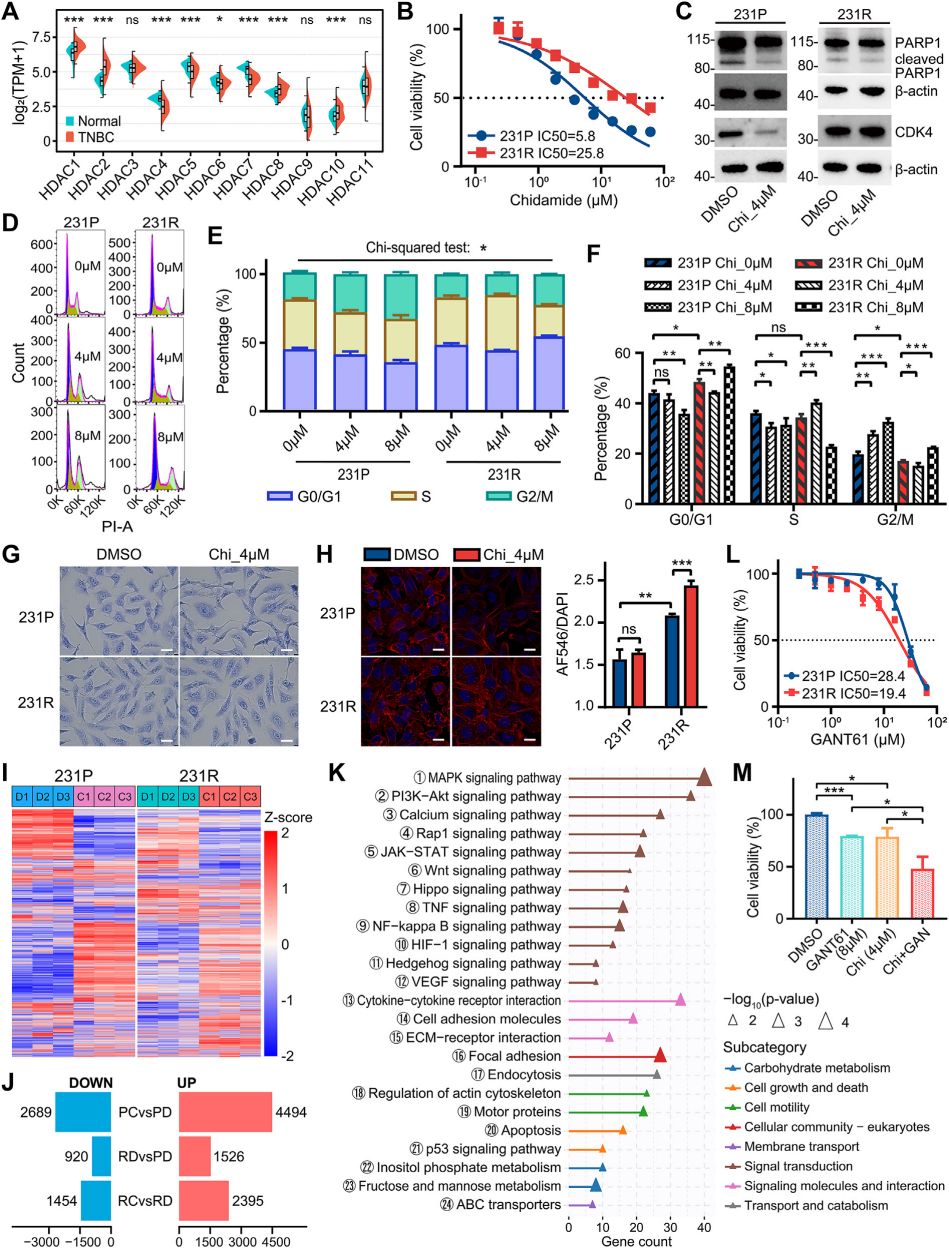
Phenotypic and molecular alterations associated with histone deacetylase (HDAC) inhibitor chidamide response and resistance in triple-negative breast cancer (TNBC). **(A)** Comparative analysis of the expression of 11 HDACs between 190 TNBC tumor tissues and 113 normal breast tissues from TCGA. Statistical significance determined by the Wilcoxon rank-sum test. **(B)** Chidamide IC50 values of MDA-MB-231 parental cells (231P) and resistant cells (231R) at 48 h. **(C)** Western blotting results showed that anti-apoptosis (full-length PARP) and proliferation (CDK4) biomarkers were down-regulated in 231P treated with chidamide compared with vehicle, while both changed little in 231R. **(D)** Cell cycle distributions by flow cytometry in 231P and 231R after treatment with 0, 4, or 8 μM chidamide for 48 h. **(E)** Chi-squared test was used to determine the phase distribution difference among groups. **(F)** After treatment with 4 or 8 μM chidamide for 48 h, the S phase population decreased significantly in 231P, while the G2/M phase population increased significantly. Instead, these phenomena were observed in 231R only after 8 μM chidamide treatment. Multiple *t*-tests were used to analyze the differences between groups, with the Holm-Sidak correction for multiple comparisons. **(G)** Giemsa staining showed distinct changes in cellular and nuclear morphology of 231P but not 231R after 24 h of treatment with 4 μM chidamide. All panels were photographed at 40 × magnification, and scale bars indicate 15 μm. **(H)** Immunofluorescence staining of F-actin (Red) and nuclei (Blue) in 231P and 231R with or without chidamide treatment (left). The relative total F-actin signal normalized by DAPI is shown alongside a barplot (right). All panels were photographed at 40 × magnification, and scale bars indicate 20 μm. **(I)** Gene expression patterns of 231P and 231R with or without chidamide treatment, shown with three biological replicates. **(J)** Number of differential genes among different groups. **(K)** ORA-based KEGG pathway enrichment analyses of 1526 genes up-regulated in 231R compared with 231P. **(L)** GLI1/GLI2 inhibitor GANT61 IC50 values of 231P and 231R at 48 h. **(M)** Chidamide plus GANT61 treatments exhibited synergistic inhibition effects in 231R cells. Bar graphs represent mean ± standard deviation. Unless otherwise stated, unpaired Student's *t* tests were used to analyze the differences between groups. ns, not significant; ∗*p* or ∗*Q* < 0.05, ∗∗*p* or ∗∗*Q* < 0.01, and ∗∗∗*p* or ∗∗∗*Q* < 0.001.

To characterize phenotypic and molecular changes associated with chidamide response and resistance in TNBC, a chidamide-resistant TNBC cell line, MDA-MB-231R (231R), was eventually established by treating wild-type cells with a year-long stepwise concentration escalation. To our knowledge, this is the first successful report of such a model in human TNBC. As anticipated, chidamide tolerance was substantially higher in 231R compared with the control MDA-MB-231P (231P), which was generated by vehicle treatment in the meantime (Fig. 1B; Fig. S3A). Similarly, there was minimal reduction in apoptosis biomarker poly (ADP-Ribose) polymerase 1 (PARP1) and cell cycle biomarker cyclin-dependent kinase 4 (CDK4) in 231R following chidamide treatment, while an obvious down-regulation was seen in 231P (Fig. 1C). Low-dose treatment disturbed the cell cycle in 231P, reducing S phase and increasing G2/M phase (Fig. 1D–F). In 231R, S phase reduction and G0/G1, G2/M phase increases occurred only with higher doses (Fig. 1D–F). Additionally, many 231P showed decreased nucleoli and heterogeneous nucleus sizes after treatment, reflecting reduced cell viability, though these changes were not statistically significant (Fig. 1G; Fig. S3B, C). In contrast, 231R exhibited narrower changes (Fig. 1G; Fig. S3B, C). Interestingly, the nuclei of 231R were significantly larger than those of 231P (Fig. S3B, C). Also, a visible morphological change occurred in lots of 231P after treatment, but not in 231R (Fig. 1G). Furthermore, chidamide induced F-actin cytoskeleton reorganization, reflecting the epithelial–mesenchymal transition, in 231P but not 231R (Fig. 1H). F-actin aggregates, signaling cell damage, were denser in 231R, regardless of treatment (Fig. 1H). Total F-actin signal was higher in 231R and further increased upon treatment (Fig. 1H). These findings highlight the distinct cellular, nuclear, and cytoskeletal structures and their alterations upon drug stimulation in TNBC cell lines with varying chidamide responsiveness.

Subsequently, RNA sequencing was utilized to explore the molecular mechanisms underlying response and resistance to chidamide. Gene expression patterns were consistent within groups while varied significantly between groups, confirming the reliability of our transcriptome data (Fig. 1I; Fig. S4A, B and Table S1, 2). In total, 7183 differential genes (|log_2_fold change| > 1, false discovery rate <0.01) were identified between chidamide and vehicle-treated 231P groups (PC *vs*. PD), including 2689 down-regulated and 4494 up-regulated genes (Fig. 1J; Fig. S4C and Table S2). Additionally, 3849 (1454 down- and 2395 up-regulated) and 2446 (920 down- and 1526 up-regulated) differential genes were found in RC versus RD and RD versus PD comparisons, respectively (Fig. 1J; Fig. S4C and Table S2). Hierarchical clustering revealed five gene clusters with distinct biological functions (Fig. S4D, E).

The Kyoto Encyclopedia of Genes and Genomes (KEGG) enrichment analysis demonstrated that down-regulated genes in the PC versus PD comparison were involved in 26 pathways (Fig. S4F and Table S3). From this, chidamide's anti-TNBC actions might be related to cell cycle progression blocking, including cell cycle arrest, p53 signaling, DNA replication, and nucleotide metabolism. This is strengthened by positive correlations between gene expression in these pathways and MKI67 in the TCGA-TNBC cohort (Fig. S4G). As for resistance-conferring mechanisms, up-regulated genes in RD versus PD comparison were involved in 24 pathways, including MAPK, phosphoinositide 3-kinase (PI3K)-protein kinase B (Akt), calcium, Ras-associated protein-1 (Rap1), Janus kinase (JAK)-signal transducers and activators of transcription (STAT), Hedgehog, Wnt, Hippo, tumor necrosis factor (TNF), nuclear factor kappa B (NFκB), hypoxia-inducible factor-1 (HIF-1), and vascular endothelial growth factor (VEGF) signals, Motor proteins, ABC transporters, *etc* (Fig. 1K and Table S4). Notably, many of them have been validated in studies on other HDACis in TNBC or other cancers (discussed in *Supplementary Materials*), highlighting the analysis's reliability. Seven genes (DNAH1, MAPK8IP3, ATP2A1, GLI2, MYH7B, TNFRSF13C, and ABCA2) were identified as potential biomarkers or drivers of chidamide resistance through correlation analyses of gene expression and chidamide sensitivity across eight TNBC cell lines from the CCLE and PRISM datasets (Fig. S4H). Research has indicated their potential roles in various cancer processes. GLI family zinc finger 2 (GLI2), a transcription factor in the Hedgehog signaling pathway, controls genes linked to cell proliferation, metastasis, therapy resistance, and stem cell maintenance in breast cancer. We confirmed that the GLI1/GLI2 inhibitor GANT61 enhanced chidamide's inhibitory effect on 231R (Fig. 1L, M). These findings enhance the clinical application prospects of HDACis, warranting further validation.

HDACis not only inhibit cancer cells but also present immunomodulatory properties. Analysis revealed significant links between HDAC expression and immune cell infiltration in the TCGA-TNBC cohort (Fig. S5A). Besides, the tumor immune dysfunction and exclusion (TIDE) algorithm indicated that over half of the HDACs, including HDAC3/4/6/7/8/9, may aid immune escape in TNBC patients (Fig. S5B). Correspondingly, higher expression of HDAC3/4/7/8/9/10 was associated with reduced immunotherapy responsiveness (Fig. S5C), suggesting their potential immunosuppressive role in TNBC. RNA sequencing data revealed significant up-regulation of immune-related genes and pathways in both 231P and 231R after chidamide treatment (Fig. S6A–C and Table S5). Additionally, 231R exhibited a slight increase in immune-related pathways compared with 231P (Fig. S6C), likely due to long-term epigenetic regulation. Pathways involving cytokines, chemokines, transforming growth factor-beta (TGF-β) family members, and their receptors were enriched in all comparisons (Fig. S6C). The pathways of interleukins and their receptors were significantly up-regulated after treatment in both 231P and 231R (Fig. S6C). Besides, T cell receptor (TCR) signaling and TNF family members and their receptors pathways were significantly enriched in PC versus PD and RC versus RD comparisons (Fig. S6C), respectively. Moreover, 231R showed increased signaling in natural killer cell cytotoxicity and antigen processing and presentation pathways compared with 231P (Fig. S6C). These results indicate that chidamide may boost anti-TNBC immunity or improve immunotherapy by modifying immune cells or other components in the tumor microenvironment.

In conclusion, our study indicated dysregulation of most HDACs in TNBC, with several linked to poor prognosis, augmented proliferation, oncogene expression, cancerogenic signal activation, immune escape, or immunotherapy resistance. Meanwhile, our RNA sequencing data and comprehensive analysis based on a stable resistant TNBC cell model unveiled a wealth of chidamide response- or resistance-associated genes and signaling pathways, offering insights into anti-tumor mechanisms and resistance-conferring determinants of HDACis in TNBC.

## CRediT authorship contribution statement

**Yanxia Zhang:** Conceptualization, Data curation, Formal analysis, Funding acquisition, Methodology, Writing – original draft. **Yunduo Liu:** Formal analysis, Investigation, Methodology, Visualization, Writing – original draft. **Mei Zhang:** Investigation, Visualization, Writing – review & editing. **Guanjie Li:** Resources, Validation, Writing – review & editing. **Qin Xiang:** Validation, Writing – review & editing. **Shunhong Wu:** Validation, Writing – review & editing. **Keping Xie:** Conceptualization, Supervision, Writing – review & editing. **Bin Xiao:** Conceptualization, Funding acquisition, Supervision, Writing – review & editing. **Linhai Li:** Conceptualization, Funding acquisition, Project administration, Writing – review & editing.

## Data availability

The public datasets analyzed during the current study are available in online repositories as described in the supplementary materials and methods. Besides, our RNA sequencing raw data have been deposited and can be accessed here: https://www.ncbi.nlm.nih.gov/bioproject/PRJNA1090785.

## Funding

This work was supported by the GuangDong Basic and Applied Basic Research Foundation (China) (No. 2021A1515111225 to Yanxia Zhang; 2024A1515013172 to Linhai Li; 2023A1515030061 to Bin Xiao) and the Science and Technology Program of Qingyuan, Guangdong, China (No. 2022KJJH027 to Linhai Li).

## Conflict of interests

Keping Xie is an associate editor for *Genes & Diseases*, and he has no involvement in the peer-review of this article and has no access to information regarding its peer-review. All authors declare that there are no competing interests.
