# Supplementary material for: Transcriptomic landscapes underlying response and resistance to HDAC inhibitor chidamide in triple-negative breast cancer
Source: Genes Dis. 2025 May 8;13(1):101676. doi: 10.1016/j.gendis.2025.101676 (PMC12466141; doi:10.1016/j.gendis.2025.101676)
Supplement: Multimedia component 1 [file mmc1.docx]

**Transcriptomic landscapes** **underlying response and resistance to HDAC inhibitor chidamide in triple-negative breast cancer**

**Supplementary Materials including:**

Abbreviations

Figures S1 to S6

Discussion

Materials and Methods

Supplementary References

**Abbreviations**

CCK-8, Cell Counting Kit-8; CCLE, Cancer Cell Line Encyclopedia; CellMinerCDB, CellMiner Cross-Database; DAPI, 4’,6-diamidino-2-phenylindole; DMSO, dimethyl sulfoxide; FC, fold change; FDR, false discovery rate; FPKM, fragments per kilobase million; GSEA, Gene Set Enrichment Analysis; GSVA, Gene Set Variation Analysis; HDAC, histone deacetylase; HDACi, HDAC inhibitor; IC50, half maximal inhibitory concentration; KEGG, Kyoto Encyclopedia of Genes and Genomes; MSigDB, Molecular Signatures Database; ORA, Over-Representation Analysis; PBS, phosphate-buffered saline; TCGA, The Cancer Genome Atlas; TIDE, Tumor Immune Dysfunction and Exclusion; TIMER, Tumor Immune Estimation Resource; TNBC, triple-negative breast cancer.

**Figures S1 to S6**


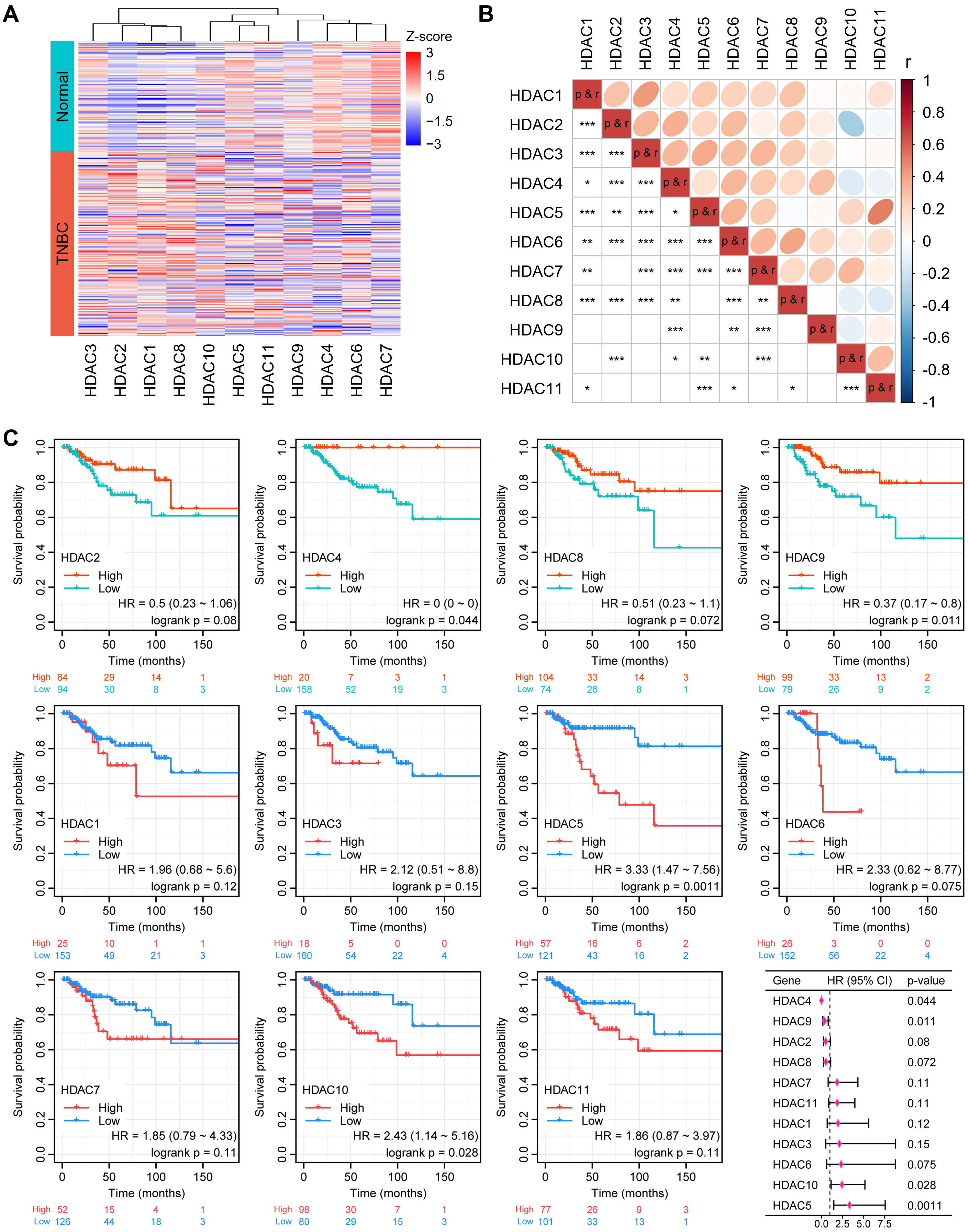


**Fig. S1** Expression pattern and prognostic value of HDACs in TCGA-TNBC cohort. **(A)** The expression pattern of HDACs in 190 TNBC tumor tissues and 113 normal breast tissues. **(B)** Pearson’s correlations among HDAC expression in TNBC tumor tissues. **(C)** Kaplan-Meier analysis of the association between HDAC expression and overall survival in TNBC cohort. Patients categorized into high and low expression groups based on optimal cut-points. *p < 0.05, **p < 0.01, ***p < 0.001, ns: not significant.


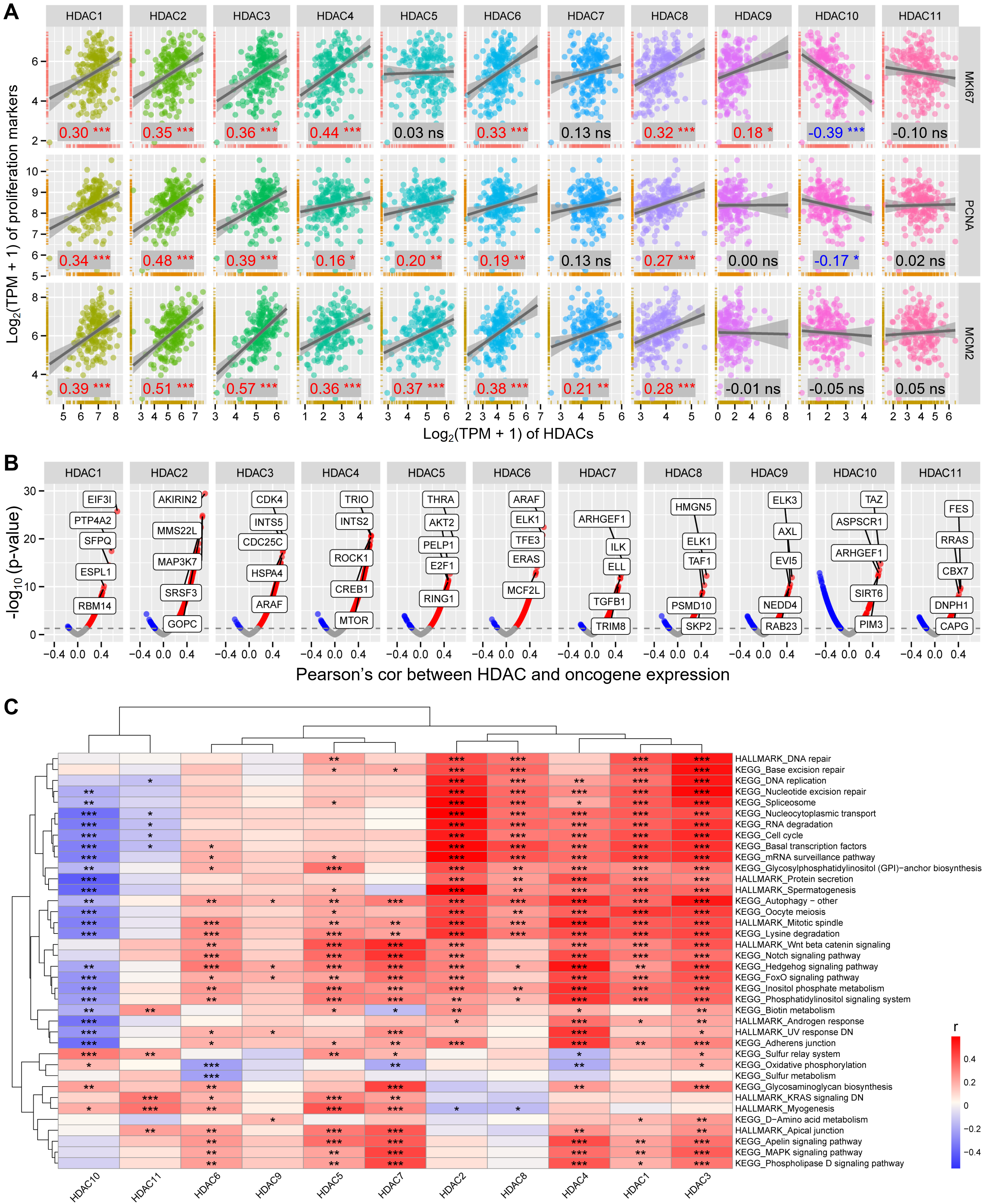


**Fig. S2** Relation between HDAC expression and proliferative activity, oncogene expression, or cancerogenic signaling pathway in TCGA-TNBC cohort. **(A)** Correlations between the expression of HDACs and those of canonical proliferation biomarkers (MKI67, PCNA, and MCM2) in TNBC tumor tissues. **(B)** Correlations between the expression of HDACs and those of 676 oncogenes in TNBC tumor tissues, with the top 5 positively correlated oncogenes for each HDAC highlighted. **(C)** Correlations analyzed between HDAC expression and the activity of 299 HALLMARK/KEGG pathways (calculated by GSVA algorithm) in TNBC tumor tissue, showing the top 5 significantly correlated pathways for each HDAC. All correlations were assessed using Pearson’s correlation. *p < 0.05, **p < 0.01, ***p < 0.001, ns: not significant.


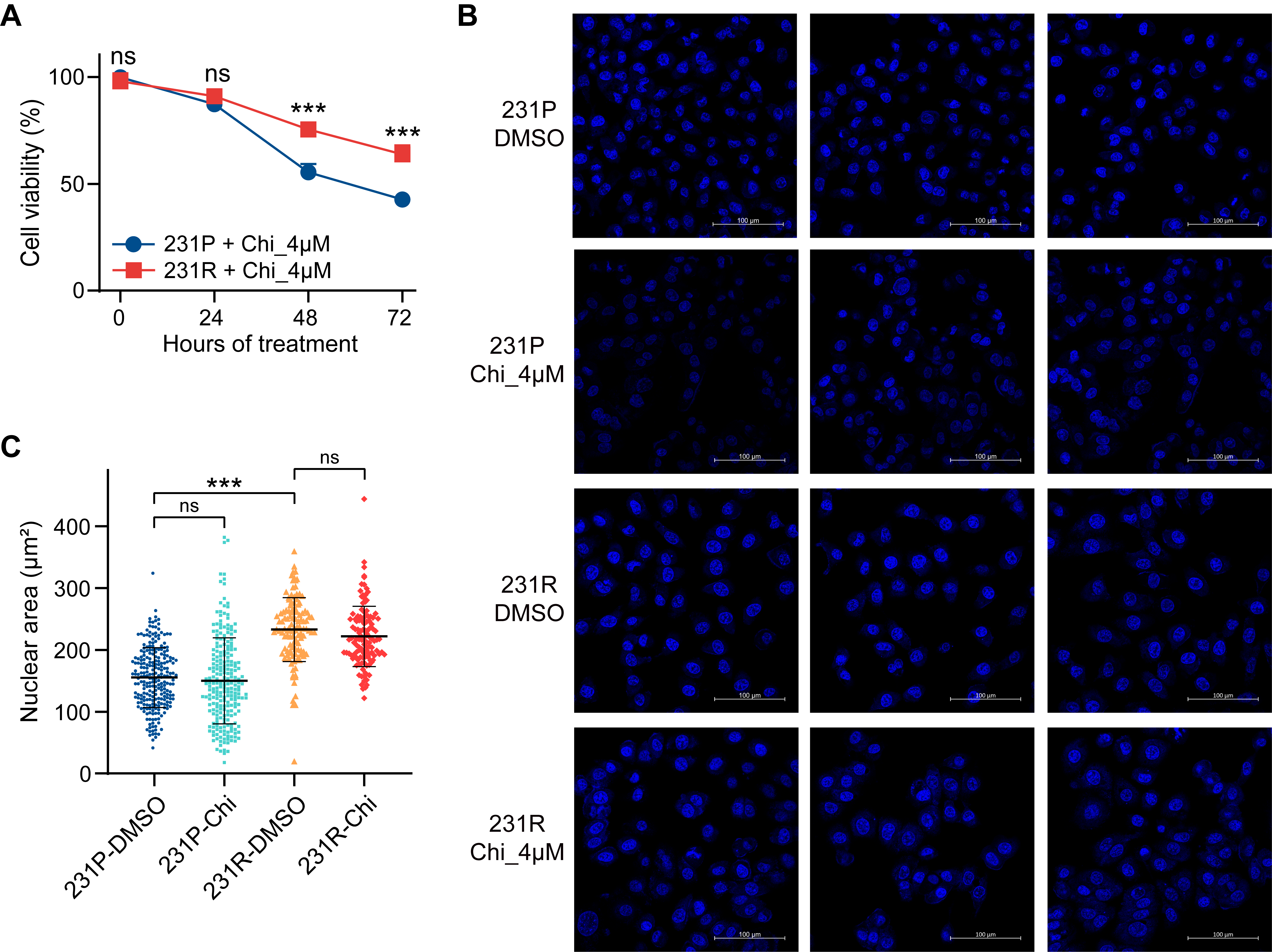


**Fig. S3** Phenotypic and molecular alterations associated with chidamide response and resistance in TNBC. **(A)** Cell viability of 231P and 231R cells after treatment with 4 μM chidamide for 24, 48, or 72 hours. **(B)** Immunofluorescence staining of nuclei (Blue) and **(C)** nuclear areas of 231P and 231R cells after treatment with 4 μM chidamide for 24 hours. Bar graphs represent mean ± SD. Statistical significance was determined by Student’s t test. ***p < 0.001, ns: not significant.


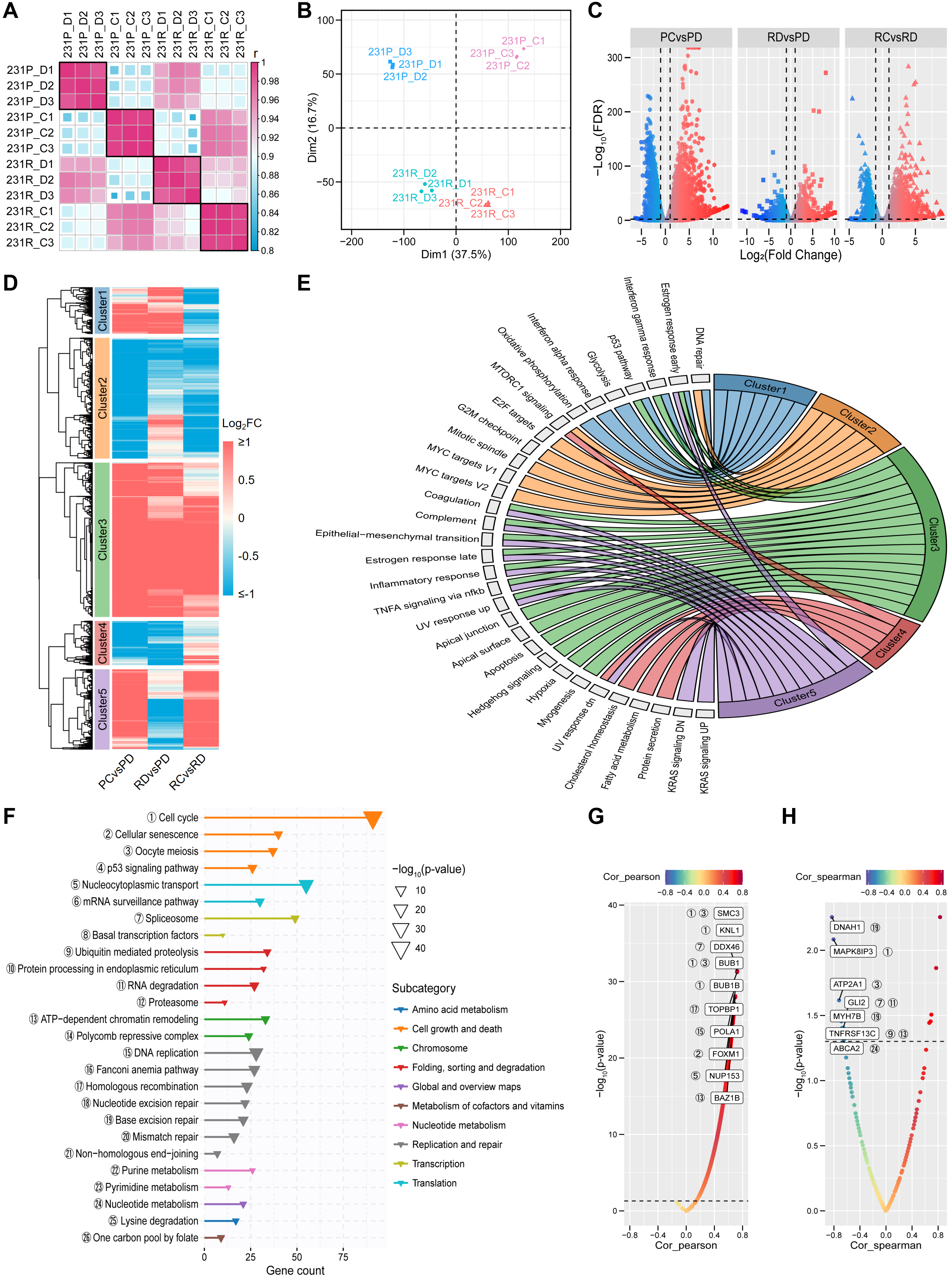


**Fig. S4** Distinct gene expression patterns or alterations upon chidamide treatment in TNBC cell lines with different chidamide responsiveness. **(A)** Pearson’s correlations between the gene expression profiles of each pair of samples. **(B)** Principal component analysis of samples based on the gene expression profiles. **(C)** Volcano plots showing differently expressed genes (|log_2_FC| > 1, FDR < 0.01). **(D)** Heat map with hierarchy cluster analysis of differently expressed genes, identifying five distinct gene clusters. Genes in cluster 1 or 3 show an upward trend in both PC vs PD and RD vs PD comparisons, while those in cluster 2 or 4 show an opposite trend. Cluster 5 genes display an upward trend in both PC vs PD and RC vs RD comparisons but a downward trend in RD vs PD comparison. **(E)** Circle plot depicting the HALLMARK gene sets enriched by different gene clusters, highlighting their involvement in various biological functions. Cluster 1 genes are related to energy metabolism, including glycolysis and oxidative phosphorylation, while cluster 2 genes are closely associated with cell proliferation and cell cycle progression. Cluster 3 genes are linked to epithelial-mesenchymal transition and immune-related pathways, with similar functions observed for cluster 5 genes. Cluster 4 genes are related to the regulation of cholesterol homeostasis and fatty acid metabolism. **(F)** ORA-based KEGG pathway enrichment analysis of 2689 genes down-regulated in 231P upon chidamide treatment. **(G)** Pearson’s correlations between the expression of 505 genes within the significantly enriched pathways in Fig. S4F and proliferation marker MKI67 in 190 TCGA-TNBC tumor tissues. The top 10 significantly and positively correlated genes were highlighted. **(H)** Spearman’s correlations between the expression of 261 genes within the significantly enriched pathways in Fig. 1K and the drug activity of chidamide (negative z-scored AUCs) in 8 TNBC cell lines based on the public CCLE and PRISM datasets. The 7 significantly and negatively correlated genes were highlighted.


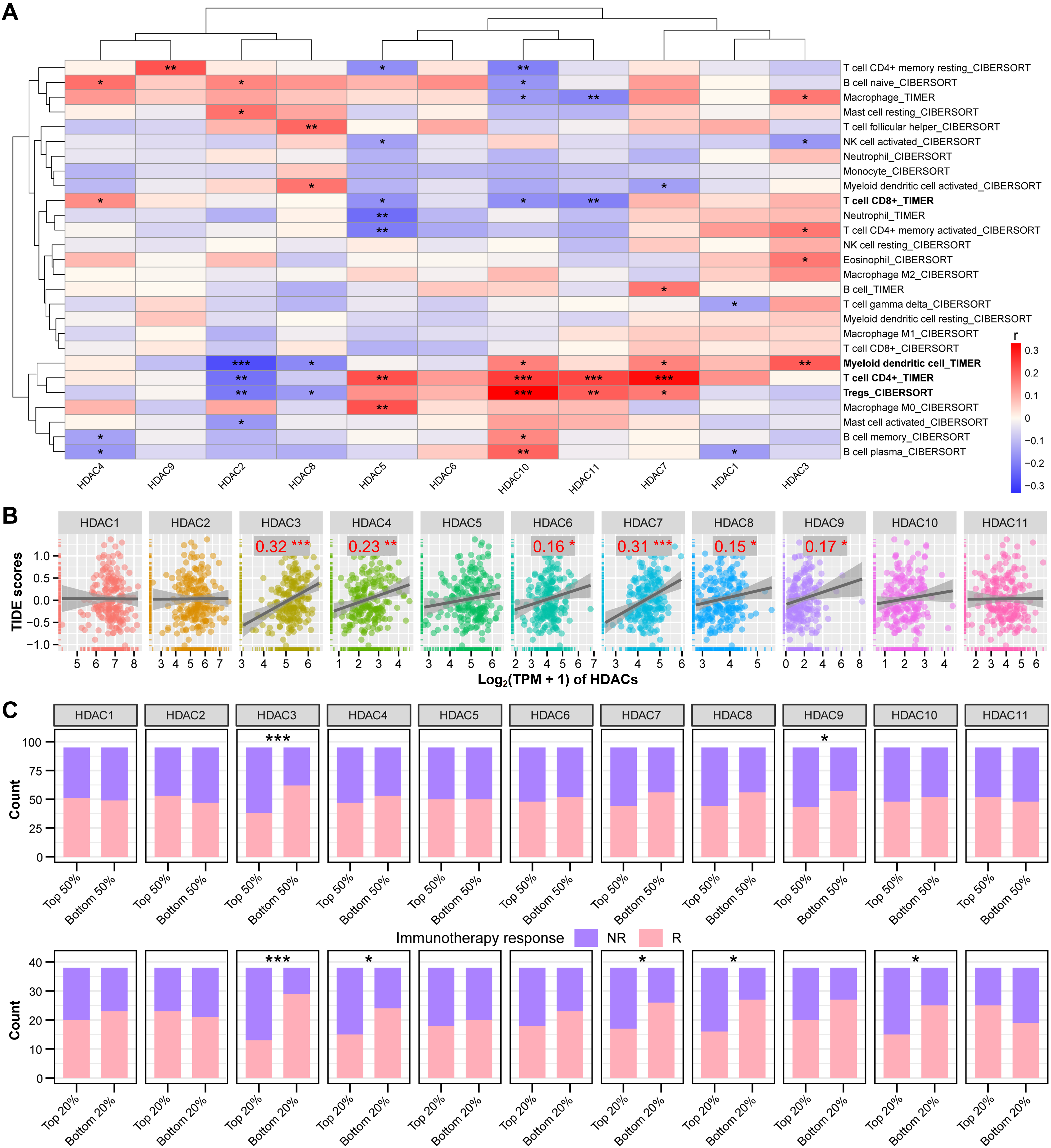


**Fig. S5** Relation between HDAC expression and immune cell infiltration as well as immunotherapy responsiveness in TCGA-TNBC cohort. **(A)** Pearson’s correlations between HDAC expression and the infiltration of immune cells (calculated using both TIMER and CIBERSORT algorithms) in TNBC tumor tissues. **(B)** Pearson’s correlations between HDAC expression and TIDE scores in TNBC tumor tissues. **(C)** Number of immunotherapy responders (R) and non-responders (NR) in high (top 50% or 20%) and low (bottom 50% or 20%) HDAC expression groups in the TCGA-TNBC cohort. Statistical significance was determined by Chi-squared test. TIDE scores and immunotherapy response data were obtained from the TIDE portal. *p < 0.05, **p < 0.01, ***p < 0.001, ns: not significant.


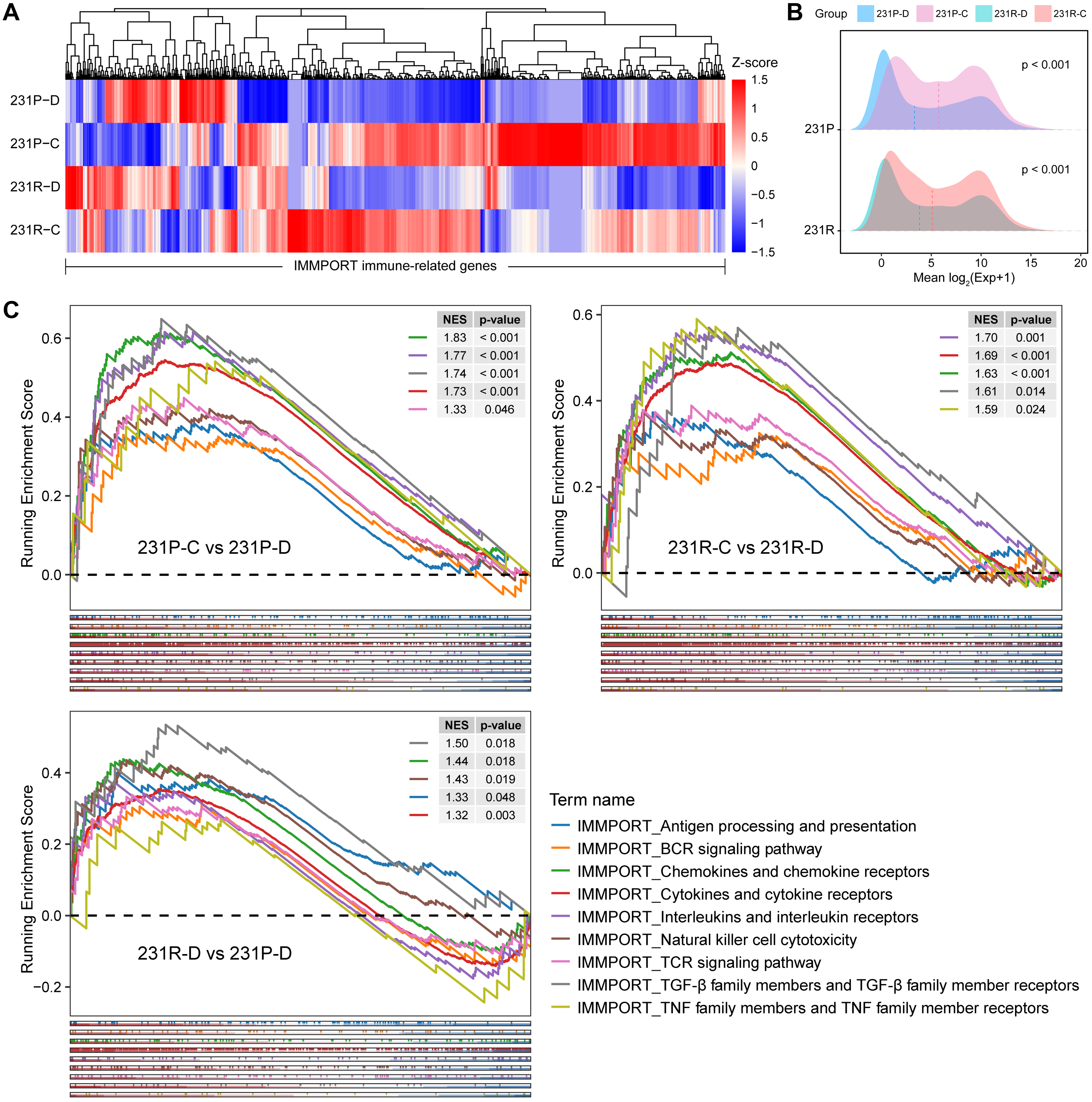


**Fig. S6** Upregulation of numerous immune-related genes and signaling pathways in TNBC cell lines following both acute and chronic chidamide treatment. **(A)** Immune-related gene expression profiles of 231P and 231R cells after treatment with vehicle or chidamide. **(B)** Overall upregulation of immune-related genes in both 231P and 231R cells after chidamide treatment as evidenced by Wilcoxon rank-sum test analysis. **(C)** The GSEA enrichment plots of 9 focused immune-related pathways.

**Discussion**

TNBC patients confront high risks of recurrence and mortality due to limited treatment options. Researchers have highlighted the intricate interplay between epigenetic dysregulation and cancer progression [1], including TNBC [2]. Dysregulated HDACs in various cancers have emerged as promising therapeutic targets [3]. This study found comprehensive evidence of HDACs’ carcinogenesis on TNBC by analyzing TCGA datasets. Particularly, overexpression of HDAC1/2/8/10 was observed in TNBC, with high levels of HDAC1/3/5/6/7/10/11 associated with poor prognosis. Additionally, positive correlations between the expression of HDAC1-HDAC9 and tumor proliferation biomarkers (MKI67, PCNA, or MCM2 [4]) underscore HDACs’ potential pro-proliferative effects on TNBC. Furthermore, elevated HDAC levels correlate with increased oncogene expression and carcinogenic pathway activity, reinforcing HDACs’ potential as drug targets for TNBC treatment [5].

Despite this promise, HDACis have shown limited efficacy as monotherapy in clinical trials for most solid tumors, including breast cancer [6]. Understanding drug response and resistance determinants is crucial for enhancing therapeutic outcomes. This study focused on chidamide, an HDAC1/2/3/10 selective inhibitor and the first globally approved HDACi for solid tumors [7]. In this study, through the development of chidamide-sensitive and -resistant TNBC cell lines, variations in representative growth and morphology characteristics related to chidamide response or resistance were demonstrated. Based on the first reported chidamide-resistant TNBC cell model, gene signatures and expression alterations associated with chidamide response and resistance were explored using RNA-seq analysis. Transcriptomic analysis revealed distinct gene expression patterns between the two cell lines, irrespective of treatment status. Notably, five gene clusters displayed diverse expression changes. Cluster 1 genes, linked to glycolysis and oxidative phosphorylation, showed upregulation in the sensitive cell line post-treatment and higher expression in the resistant cell line compared to the sensitive cell line without treatment, suggesting metabolic adaptations in TNBC cells post short- or long-term chidamide treatment, which have been reported in other solid tumors treated with HDACis [8, 9]. Cluster 2 genes, consistently down-regulated in all comparisons, were cell proliferation and cycle progression associated, reflecting chidamide’s principal anti-TNBC mechanisms. Finally, cluster 3 genes, involved in epithelial-mesenchymal transition and immune-related pathways, were consistently upregulated in all comparisons, suggesting the potent directions for evaluating or enhancing HDACi treatment.

Enrichment analyses of total differential genes showed 26 and 24 potential KEGG pathways possibly involved with chidamide response and resistance, respectively. These results indicated that chidamide’s action mechanisms on TNBC cells may be linked to cell cycle progression blocking, such as cell cycle arrest, p53 signaling, DNA replication, and nucleotide metabolism, either directly or indirectly. Understanding these mechanisms underlying drug action presents a fertile area for future research. As for mechanisms of chidamide resistance, enrichment terms prompted the potential involvement of multiple pathways, including MAPK, PI3K-Akt, Calcium, Rap1, JAK-STAT, Hedgehog, Wnt, Hippo, TNF, NF-kappa B, HIF-1, and VEGF signals, as well as motor proteins and ABC transporters. Notably, MAPK signaling pathway can protect lung cancer cells from HDACi LBH589-induced cell death [10]. Activation of MAPK signal during HDACi CUDC-907 treatment appeared in TNBC cells as well [11]. In addition, feedback activation of PI3K-Akt [12], JAK-STAT3 [13, 14], and Hedgehog [15] signals have been linked to therapeutic resistance to HDACi panobinostat or vorinostat in TNBC cells. Interestingly, we also preliminarily found the potential of Hedgehog signal inhibitor GANT61 and chidamide in combination in chidamide-resistant TNBC cells. Activation of NF-kappa B [16, 17], HIF-1 [18], and VEGF [18] signals have also been reported as resistance drivers of HDACi vorinostat or trichostatin A in other cancer types, and thereby extending these mechanisms to HDACi-resistant TNBC is indeed warranted. Motor proteins, essential for intracellular transport and communication, as well as microtubule dynamics regulation among other functions [19], are also involved. The combination of HDACi OBP-801 and eribulin, a microtubule dynamics inhibitor, has demonstrated synergistic inhibition of TNBC cell growth [20], highlighting the significance of exploring the association between chidamide resistance and motor proteins. What’s more, careful attention should be given to ABC transporters, which play pivotal roles in eliciting drug efflux, representing a key factor driving intrinsic or acquired resistance of tumor cells to a wide range of antineoplastic drugs [21]. The upregulation of ABC transporters following HDACi treatment has been observed in several studies [22], although it is less explored in TNBC. Overall, cross-activation of oncogenic signals is likely a crucial mechanism driving resistance to anti-cancer therapies, including HDACis such as chidamide. Future exploration of these pathways and their interactions with chidamide treatment could offer valuable insights for developing effective strategies to enhance HDACi treatment in TNBC.

Finally, it’s worth noting the impact of HDACs on anti-tumor immunity in TNBC. Our analysis of TCGA data revealed intrinsically correlations between HDAC expression and immune cell infiltration in the TME. What’s more, the expression of several HDACs, including HDAC3/4/6/7/8/9, showed positive correlations with immune escape, and upregulation of HDAC3/4/7/8/9/10 appeared to incur immunotherapy resistance in TNBC. Echoing these findings, our RNA-seq data analysis demonstrated significant upregulation of several important immune-related pathways following chidamide treatment, suggesting the potential roles of chidamide as an immunostimulant for TNBC. Indeed, accumulating evidence substantiates this concept, as HDACi treatment has been demonstrated to enhance efficacy in various cancers, including breast cancer, through mechanisms related to immune modulation. These mechanisms include the alteration of tumor-associated antigens, the promotion of immune checkpoint agonists, proteins governing tumor immune recognition and antigen processing, the expression of tumor inflammation-related genes, chemokines, CD8+ T cells and T cell response, M1 macrophages, natural killer cells, and reduction of myeloid-derived suppressor cells, among other effects [23]. From this study, we believe that chidamide may boost anti-TNBC immunity or improve immunotherapy effectiveness by multiple molecular mechanisms derived from tumor cells as describe in the main text and ultimately modify immune cells and tumor microenvironment, whereas the mechanisms warrant further exploration in TNBC. Additionally, a modest upregulation of immune-related pathways was observed in chidamide-resistant TNBC cells compared to parental cells. This could be attributed to long-term epigenetic regulation rather than a chidamide resistance-conferring mechanism, and further research is necessary to solidify this.

However, it’s important to acknowledge some limitations in this study. For instance, only one TNBC cell line model was utilized, and further exploration of the morphology and molecular findings with additional models is necessary. So we will keep working on it in the future. Furthermore, although some chidamide response and resistance mechanisms get supports by previous excellent researches involving HDACis and solid tumors, additional well-designed experiments are required to validate or extend our initial observations for TNBC.

In conclusion, a stable chidamide-resistant TNBC cell model was successfully developed. To our knowledge, this is the first successful report of such a model in human TNBC. Comprehensive analysis of transcriptomic data has unveiled a wealth of potentially altered genes and signaling pathways, offering valuable resources for gaining insights into the mechanisms underlying HDACi response and resistance in TNBC.

**Materials and methods**

**Cell culture and growth conditions**

The human TNBC cell line MDA-MB-231 was obtained from the Shanghai Cell Bank of the Chinese Academy of Sciences. Cells were cultured in DMEM high glucose medium (Gibco, C11995500CP) containing L-glutamine, 4.5 g/L D-glucose, 110 mg/L sodium pyruvate, 10% fetal bovine serum (ExCell Bio, FSP500), and 1% penicillin/streptomycin (Gibco, 15140-122). Cultures were maintained at 37°C in a humidified atmosphere with 5% CO_2_.

**Reagents and antibodies**

Chidamide (Selleck, S8567), also known as Tucidinostat, was prepared as a 25 mM stock solution in dimethyl sulfoxide (DMSO, Sigma-Aldrich, D2650) and aliquoted into microcentrifuge tubes. GANT61 (Selleck, S8075), was dissolved in ethanol of 25 mM. All stock tubes were stored at -80 ℃, while working tubes were kept at -20 ℃ for up to one month. For western blot analysis, antibodies were used against β-actin (Absin, abs132001, 1:5000), CDK4 (Absin, abs155981, 1:1000), PARP1 (Proteintech, 66520-1-Ig, 1:5000).

**Drug-resistant cell line generation and drug resistance maintenance**

Cells were seed in 10-cm dishes one day before treatment. When cell cultures reached 30% confluence, a complete medium with 0.5/1/2/4/6/8 μM of chidamide or an equal volume of vehicle was added. After 48 hours, the medium was replaced with drug-free medium to allow recovery. Cells were subcultured in the drug-free medium at 90% confluence and re-exposed to the drug in the next cycle. This process was repeated for six to eight cycles per dose until cell viability showed minimal or no decrease post-treatment. Increased resistance in the drug-treated group was confirmed compared to the vehicle group. Resistant (231R) and parental (231P) cells were then exposed to 8 μM of chidamide or vehicle monthly to maintain their distinct drug responsiveness.

**Cell viability assays**

Cells were plated in a 96-well plate at a density of 3000-5000 cells per well in at least triplicate one day before treatment. Following indicated treatment, cell viability was assessed using the Cell Counting Kit-8 (CCK-8) (GLPBIO, CCK801) according to the manufacturer’s instruction. Optical density (OD) was measured at 450 nm. Cell viability was calculated relative to the vehicle group. Dose-response curves were plotted, and half maximal inhibitory concentration (IC50) values were determined by fitting dose-response curves in GraphPad Prism 7.04.

**Western blot analysis**

Rinsed cells were lysed using lysis buffer, and protein concentrations were determined using the BCA Protein Assay kit (Beyotime, P0011). Equal amounts of cell lysate were then subjected to SDS-PAGE and transferred onto a PVDF membrane (Millipore). The membranes were incubated with primary antibodies overnight at 4 °C, followed by incubation with appropriated diluted secondary antibodies conjugated with horseradish peroxidase (Abcam ab205718 or Cell Signal Techology #7076). Immunoreactive bands were visualized using chemoluminescence detection reagent (Millipore, WBKLS0100).

**Cell cycle phase distribution measurement**

Cells were harvested and processed for staining and detection using the cell cycle and apoptosis analysis kit (Beyotiome, C1052). Briefly, cell cultures were trypsinized and washed with pre-chilled phosphate-buffered saline (PBS). Afterward, cells were fixed in pre-chilled 70% ethanol for 12-24 hours, followed by washing with pre-cooling PBS. The fixed cells were then stained with propidium staining solution for 30 min at room temperature and analyzed using flow cytometry (BD FACSMelody). Cell cycle phase distribution was determined using FlowJo v10.8.1.

**Morphological observations**

Cells were seeded at 2.5×10^5 cells per well in a 6-well plate one day before treatment. Following indicated treatment, cells were washed twice with PBS, fixed with 4% paraformaldehyde for 10 minutes at 37°C, and then stained with modified Giemsa staining solution (Beyotime, C0131) at a concentration of 20× for 35 minutes. After washing and drying, stained cells were imaged using a microscope-mounted camera. Cellular and nuclear perimeters were measured using ImageJ. Filamentous actin (F-actin) was visualized using tetramethylrhodamine isothiocyanate-labeled phalloidin (Solarbio, CA1610). Approximately 30000 cells were seeded in each glass-bottom dish (Cellvis, D35-14-1-N) designed for imaging using a laser scanning confocal microscope one day before treatment. Following indicated treatment, cells were fixed with 4% paraformaldehyde for 10 minutes at 37°C, permeabilized with 0.5% Triton X-100 for 5 minutes, and then incubated with a 100 nM phalloidin solution for 30 minutes at room temperature under light-protected conditions. Additionally, nuclei were labeled with 4’,6-diamidino-2-phenylindole (DAPI) at a final concentration of 1 μg/ml. Specimens were observed and photographed using a laser scanning confocal microscope. To ascertain the size of the cell nuclei, approximately 30000 cells were seeded per glass-bottom dish one day prior drug treatment. Following 24 hours of drug exposure, cells were stained with Hoechst 33342 dye at 37°C for 30 minutes within a cell culture incubator. Subsequently, the cells underwent three washes with PBS and were fixed with 70% ethanol for 30 minutes. After three additional PBS washes, the samples were imaged using a laser confocal microscope. The nuclear area was measured using ImageJ software, and subjected to statistical analysis using GraphPad Prism version 7.04.

**RNA library preparation and sequencing**

Cells were exposed to 8 μM chidamide or vehicle for 24 hours in 10-cm dishes. Following treatment and sample collection, over 6×10^6 cells per sample were rapidly frozen in liquid nitrogen. Total RNA was isolated using TRIzol reagents (Thermo Fisher, 15596018). Messenger RNA (mRNA) extraction was performed using poly-T oligo-attached magnetic beads and fragmented with divalent cations at an elevated temperature. First-strand cDNA synthesis was carried out using random hexamer primers and RNase H- M-MuLV Reverse Transcriptase. Second-strand cDNA synthesis was performed using DNA Ploymerase I and RNase H. Exonuclease and polymerase converted remaining overhangs into blunt ends. The 3’ ends of DNA fragments were adenylated, and adaptors were ligated. The library fragments were purified using the AMPure XP system (Beckman Coulter, Beverly, USA), with a preference for length of 250-300 bp. The initial library was amplified via PCR using Phusion High-Fidelity DNA Polymerase and universal or index primers. The resulting products were purified using the AMPure XP system and assessed on the Agilent Bioanalyzer 5400 system. The cBot Cluster Generation System and Illumina TruSeq PE Cluster Kit v3-cBot-HS clustered the samples. Library were sequenced were on the Illumina NovaSeq 6000 platform, generating paired-end reads of 150 bp.

**Gene expression quantification and differential analysis**

Raw data in FASTQ format underwent preprocessing to generate clean reads using fastp v0.23.1, filtering out reads containing adapters, poly-N sequences, or of low quality. Subsequently, clean reads were mapped to GRCh38 reference genome with HISAT2 v2.0.5, and reads mapped to each gene were counted with featureCounts v1.5.0-p3. Gene expression levels were quantified as fragments per kilobase million (FPKM), considering both gene length and read count. The “DESeq2” v1.29.0 R package was employed for differential expression analysis. P values were adjusted to control the false discovery rate (FDR) using the Benjamini and Hochberg method. Genes with an FDR < 0.01 and an absolute log2 fold change (FC) greater than 1 were considered differentially expressed. The gene expression quantification matrix in FPKM is provided in Table S1, while the comparative results, including log_2_FC, p values, and FDR are presented in Table S2.

**Acquisition of** **publicly available data for TNBC patients and cell lines**

Transcriptomic data with survival information for TNBC patients were obtained from TCGA portal (https://www.cancer.gov/tcga/) [24]. Transcriptomic data for eight TNBC cell lines were acquired from Cancer Cell Line Encyclopedia (CCLE, https://sites.broadinstitute.org/ccle/) [25]. Drug activity data, represented as negative z-scored area under the curve (AUCs), for chidamide were sourced from the PRISM project [26] using CellMiner Cross-Database (CellMinerCDB, https://discover.nci.nih.gov/rsconnect/cellminercdb/) [27].

**Acquisition of reference gene sets and oncogene list**

HALLMARK gene sets were sourced from Molecular Signatures Database (MSigDB, https://www.gsea-msigdb.org/gsea/msigdb/) [28]. Kyoto Encyclopedia of Genes and Genomes (KEGG) [29] gene sets were extracted using the “KEGGREST” R package, and excluding those in the “Human Diseases” or “Organismal Systems” categories. Immune-related gene sets were obtained from the ImmPort portal (https://www.immport.org/) [30]. The oncogenes list was downloaded from the ONGene portal (http://ongene.bioinfo-minzhao.org/) [31].

**Pathway enrichment analyses (ORA,** **GSEA, and GSVA)**

Over-Representation Analysis (ORA) and Gene Set Enrichment Analysis (GSEA) [32] were performed using the “clusterProfiler” R package [33] to identify significant pathway enrichment. Gene Set Variation Analysis (GSVA) was conducted using the “GSVA” R package [34] to assess the overall activity of particular pathways. The enrichment analysis results of our sequencing data are shown in Tables S3 to S5.

**Survival analysis**

To investigate HDACs’ prognostic significance in TNBC patients, TCGA-TNBC cohort data were used for survival analyses. Individuals with < 1 month overall survival were excluded. The “surv_cutpoint” function from “survminer” R package determined the optimal HDAC cutpoints, stratifying patients into high and low expression groups. Kaplan-Meier curves and log-rank tests were performed using “survminer” and “survival” R packages.

**Analysis of immune infiltration and immunotherapy response**

The Tumor Immune Estimation Resource (TIMER, http://timer.cistrome.org/) portal [35] assessed the infiltration of representative immune cells using TIMER and Cell-type Identification By Estimating Relative Subsets Of RNA Transcripts (CIBERSORT) [36] algorithms. Tumor Immune Dysfunction and Exclusion (TIDE, http://tide.dfci.harvard.edu/) [37] estimated TIDE scores and predicted immunotherapy responsiveness based on gene expression profiles. A higher TIDE score indicates a higher probability of immune escape, thus suggesting a lower responsiveness to immunotherapy.

**Statistical analysis**

Differences between groups were examined by Wilcoxon rank-sum test (implemented via the “wilcox.test” function in R) or by Student’s t test (conducted either in R using “t.test” function or in GraphPad Prism, with Holm-Sidak correction applied for multiple t test comparisons when required). Correlations were evaluated with Pearson’s or Spearman’s correlations using the “cor.test” function in R. Distribution analyses were conducted using the Chi-squared test in GraphPad Prism or the “chisq.test” function in R. Statistical significance was set at p or adjusted p (Q) < 0.05 unless otherwise stated.

**Supplementary References**

1. Singh M, Kumar V, Sehrawat N, Yadav M, Chaudhary M, Upadhyay SK, et al. Current paradigms in epigenetic anticancer therapeutics and future challenges. Seminars in Cancer Biology. 2022;83:422-440.

2. Zolota V, Tzelepi V, Piperigkou Z, Kourea H, Papakonstantinou E, Argentou Mu I, et al. Epigenetic alterations in triple-negative breast cancer-the critical role of extracellular matrix. Cancers (Basel). 2021;13(4):713.

3. Neganova ME, Klochkov SG, Aleksandrova YR, Aliev G. Histone modifications in epigenetic regulation of cancer: Perspectives and achieved progress. Seminars in Cancer Biology. 2022;83:452-471.

4. Sun Y, Cheng Z, Liu S. MCM2 in human cancer: functions, mechanisms, and clinical significance. Mol Med. 2022;28(1):128.

5. Ramaiah MJ, Tangutur AD, Manyam RR. Epigenetic modulation and understanding of HDAC inhibitors in cancer therapy. Life Sci. 2021;277:119504.

6. Liang T, Wang F, Elhassan RM, Cheng Y, Tang X, Chen W, et al. Targeting histone deacetylases for cancer therapy: Trends and challenges. Acta Pharmaceutica Sinica B. 2023;13(6):2425-2463.

7. Jiang Z, Li W, Hu X, Zhang Q, Sun T, Cui S, et al. Tucidinostat plus exemestane for postmenopausal patients with advanced, hormone receptor-positive breast cancer (ACE): a randomised, double-blind, placebo-controlled, phase 3 trial. The Lancet Oncology. 2019;20(6):806-815.

8. Nguyen TTT, Zhang Y, Shang E, Shu C, Torrini C, Zhao J, et al. HDAC inhibitors elicit metabolic reprogramming by targeting super-enhancers in glioblastoma models. Journal of Clinical Investigation. 2020;130(7):3699-3716.

9. Bishayee K, Nazim UM, Kumar V, Kang J, Kim J, Huh SO, et al. Reversing the HDAC-inhibitor mediated metabolic escape in MYCN-amplified neuroblastoma. Biomed Pharmacother. 2022;150:113032.

10. Yu C, Friday BB, Lai J-P, McCollum A, Atadja P, Roberts LR, et al. Abrogation of MAPK and Akt signaling by AEE788 synergistically potentiates histone deacetylase inhibitor-induced apoptosis through reactive oxygen species generation. Clinical Cancer Research. 2007;13(4):1140-1148.

11. Li Z-J, Hou Y-J, Hao G-P, Pan X-X, Fei H-R, Wang F-Z. CUDC-907 enhances TRAIL-induced apoptosis through upregulation of DR5 in breast cancer cells. Journal of Cell Communication and Signaling. 2020;14(4):377-387.

12. Lyu H, Hou D, Liu H, Ruan S, Tan C, Wu J, et al. HER3 targeting augments the efficacy of panobinostat in claudin-low triple-negative breast cancer cells. NPJ Precis Oncol. 2023;7(1):72.

13. Zeng H, Qu J, Jin N, Xu J, Lin C, Chen Y, et al. Feedback activation of leukemia inhibitory factor receptor limits response to histone deacetylase inhibitors in breast cancer. Cancer Cell. 2016;30(3):459-473.

14. Zhao C, Zhang Y, Zhang Jg, Li S, Liu M, Geng Y, et al. Discovery of novel fedratinib-based HDAC/JAK/BRD4 triple inhibitors with remarkable antitumor activity against triple negative breast cancer. Journal of Medicinal Chemistry. 2023;66(20):14150-14174.

15. Wang X, Xu J, Sun Y, Cao S, Zeng H, Jin N, et al. Hedgehog pathway orchestrates the interplay of histone modifications and tailors combination epigenetic therapies in breast cancer. Acta Pharmaceutica Sinica B. 2023;13(6):2601-2612.

16. Zheng L, Fu Y, Zhuang L, Gai R, Ma J, Lou J, et al. Simultaneous NF‐κB inhibition and E‐cadherin upregulation mediate mutually synergistic anticancer activity of celastrol and SAHA in vitro and in vivo. International Journal of Cancer. 2014;135(7):1721-1732.

17. Karthik S, Sankar R, Varunkumar K, Anusha C, Ravikumar V. Blocking NF-κB sensitizes non-small cell lung cancer cells to histone deacetylase inhibitor induced extrinsic apoptosis through generation of reactive oxygen species. Biomedicine & Pharmacotherapy. 2015;69:337-344.

18. Lee J-W, Yang DH, Park S, Han H-K, Park J-W, Kim BY, et al. Trichostatin A resistance is facilitated by HIF-1α acetylation in HeLa human cervical cancer cells under normoxic conditions. Oncotarget. 2017;9(2):2035-2049.

19. Hassan Ibrahim I, Balah A, Gomaa Abd Elfattah Hassan A, Gamal Abd El-Aziz H. Role of motor proteins in human cancers. Saudi J Biol Sci. 2022;29(12):103436.

20. Ono H, Sowa Y, Horinaka M, Iizumi Y, Watanabe M, Morita M, et al. The histone deacetylase inhibitor OBP-801 and eribulin synergistically inhibit the growth of triple-negative breast cancer cells with the suppression of survivin, Bcl-xL, and the MAPK pathway. Breast Cancer Research and Treatment. 2018;171(1):43-52.

21. Sajid A, Rahman H, Ambudkar SV. Advances in the structure, mechanism and targeting of chemoresistance-linked ABC transporters. Nature Reviews Cancer. 2023;23(11):762-779.

22. Ni X, Li LI, Pan G. HDAC inhibitor-induced drug resistance involving ATP-binding cassette transporters (Review). Oncology Letters. 2015;9(2):515-521.

23. Lian B, Chen X, Shen K. Inhibition of histone deacetylases attenuates tumor progression and improves immunotherapy in breast cancer. Front Immunol. 2023;14:1164514.

24. Hutter C, Zenklusen JC. The cancer genome atlas: Creating lasting value beyond its data. Cell. 2018;173(2):283-285.

25. Barretina J, Caponigro G, Stransky N, Venkatesan K, Margolin AA, Kim S, et al. The Cancer Cell Line Encyclopedia enables predictive modelling of anticancer drug sensitivity. Nature. 2012;483(7391):603-607.

26. Yu C, Mannan AM, Yvone GM, Ross KN, Zhang Y-L, Marton MA, et al. High-throughput identification of genotype-specific cancer vulnerabilities in mixtures of barcoded tumor cell lines. Nature Biotechnology. 2016;34(4):419-423.

27. Luna A, Elloumi F, Varma S, Wang Y, Rajapakse Vinodh N, Aladjem MI, et al. CellMiner Cross-Database (CellMinerCDB) version 1.2: Exploration of patient-derived cancer cell line pharmacogenomics. Nucleic Acids Research. 2021;49(D1):D1083-D1093.

28. Subramanian A, Tamayo P, Mootha VK, Mukherjee S, Ebert BL, Gillette MA, et al. Gene set enrichment analysis: A knowledge-based approach for interpreting genome-wide expression profiles. Proceedings of the National Academy of Sciences. 2005;102(43):15545-15550.

29. Kanehisa M, Furumichi M, Sato Y, Ishiguro-Watanabe M, Tanabe M. KEGG: integrating viruses and cellular organisms. Nucleic Acids Research. 2021;49(D1):D545-D551.

30. Bhattacharya S, Dunn P, Thomas CG, Smith B, Schaefer H, Chen J, et al. ImmPort, toward repurposing of open access immunological assay data for translational and clinical research. Sci Data. 2018;5(1):180015.

31. Liu Y, Sun J, Zhao M. ONGene: A literature-based database for human oncogenes. Journal of Genetics and Genomics. 2017;44(2):119-121.

32. Mootha VK, Lindgren CM, Eriksson K-F, Subramanian A, Sihag S, Lehar J, et al. PGC-1α-responsive genes involved in oxidative phosphorylation are coordinately downregulated in human diabetes. Nature Genetics. 2003;34(3):267-273.

33. Wu T, Hu E, Xu S, Chen M, Guo P, Dai Z, et al. clusterProfiler 4.0: A universal enrichment tool for interpreting omics data. Innovation (Camb). 2021;2(3):100141.

34. Hanzelmann S, Castelo R, Guinney J. GSVA: gene set variation analysis for microarray and RNA-seq data. BMC Bioinformatics. 2013;14(1):7.

35. Liu XS, Li B, Chen Q, Li J, Cohen D, Zeng Z, et al. TIMER2.0 for analysis of tumor-infiltrating immune cells. Nucleic Acids Research. 2020;48(W1):W509-W514.

36. Chen B, Khodadoust MS, Liu CL, Newman AM, Alizadeh AA. Profiling tumor infiltrating immune cells with CIBERSORT. Methods Mol Biol. 2018;1711:243-259.

37. Jiang P, Gu S, Pan D, Fu J, Sahu A, Hu X, et al. Signatures of T cell dysfunction and exclusion predict cancer immunotherapy response. Nature Medicine. 2018;24(10):1550-1558.
